# Comparative Plasma and Interstitial Fluid Pharmacokinetics of Meloxicam, Flunixin, and Ketoprofen in Neonatal Piglets

**DOI:** 10.3389/fvets.2020.00082

**Published:** 2020-02-20

**Authors:** Emma Nixon, Glen W. Almond, Ronald E. Baynes, Kristen M. Messenger

**Affiliations:** ^1^Department of Population Health and Pathobiology, College of Veterinary Medicine, North Carolina State University, Raleigh, NC, United States; ^2^Department of Molecular Biomedical Sciences, College of Veterinary Medicine, North Carolina State University, Raleigh, NC, United States

**Keywords:** piglet, pain, welfare, castration, NSAID, pharmacokinetics, interstitial fluid

## Abstract

Piglet castration and tail-docking are routinely performed in the United States without analgesia. Pain medications, predominately non-steroidal anti-inflammatory drugs, are used in the EU/Canada to decrease pain associated with processing and improve piglet welfare, however, past studies have shown the efficacy and required dose remain controversial, particularly for meloxicam. This study assessed the pharmacokinetics of three NSAIDs (meloxicam, flunixin, and ketoprofen) in piglets prior to undergoing routine castration and tail-docking. Five-day-old male piglets (8/group) received one of 3 randomized treatments; meloxicam (0.4 mg/kg), flunixin (2.2 mg/kg), ketoprofen (3.0 mg/kg). Two hours post-dose, piglets underwent processing. Drug concentrations were quantified in plasma and interstitial fluid (ISF) and pharmacokinetic parameters were generated by non-compartmental analysis. Time to peak concentration (T_max_) of meloxicam, flunixin, and S(–)-ketoprofen in plasma were 1.21, 0.85, and 0.59 h, compared to 2.81, 3.64, and 2.98 h in the ISF, respectively. The apparent terminal half-life of meloxicam, flunixin and S(–)-ketoprofen were 4.39, 7.69, and 3.50 h, compared to 11.26, 16.34, and 5.54 h, respectively in the ISF. If drug concentrations in the ISF are more closely related to efficacy than the plasma, then the delay between the Tmax in plasma and ISF may be relevant to the timing of castration in order to provide the greatest analgesic effect.

## Introduction

Consumers are becoming increasingly concerned with welfare issues associated with castration and tail-docking of piglets, and this has prompted an increase in the investigation of pain medications to provide analgesia during these procedures. In the United States, husbandry procedures such as castration, tail-docking, teeth clipping, ear notching/tagging, and injections are collectively referred to as “processing” of piglets. Commercial farms in the United States routinely perform processing procedures (including castration and tail docking) without anesthesia or analgesia, despite the fact that these procedures are painful and distressing to piglets (1–7). The intent of surgical castration is to reduce aggression among male pigs as well as reduce the incidence of boar taint, an offensive odor or taste detected in pork obtained from intact male pigs following puberty. Tail docking preemptively aims to prevent tail biting. While currently there is no requirement for the provision of analgesics for piglets in the US, legislation in the EU and Canada requires that surgical castration of piglets should be performed with anesthesia and/or analgesia (8, 9). Additionally, routine tail docking is forbidden in the EU and may only be performed when there is evidence that tail biting has occurred (10).

Non-steroidal anti-inflammatory drugs (NSAIDs) are among the most commonly studied treatments for processing-associated pain (11, 12), however, there are conflicting data supporting their use at castration or tail-docking. Particularly, there is conflicting data supporting the use of meloxicam at the European label dose of 0.4 mg/kg (13–16). There are no pharmacokinetic studies of meloxicam in piglets <1 week-of-age, which is surprising given the larger number of studies assessing the efficacy of meloxicam for pain-alleviation following castration, compared to other NSAIDs. Studies have assessed the plasma pharmacokinetics of meloxicam administered intramuscularly at 1.0 mg/kg in 8-day-old piglets (17), at 0.6 mg/kg in 2-week-old piglets (18), and at 0.4 mg/kg intravenously in pigs 16–23 days old (19). None of these studies assessed the EU label dose of 0.4 mg/kg administered intramuscularly, and none assessed meloxicam in piglets <1 week old. Piglets are typically castrated between 3 and 7 days of age, and age may affect the pharmacokinetics of these NSAIDs (19). Flunixin plasma pharmacokinetics were previously described following an intravenous dose of 2.2 or 4.4 mg/kg in piglets 6–8 days old (20), but have not been described in neonatal piglets following intramuscular administration. Ketoprofen plasma pharmacokinetics parameters were reported following an intramuscular dose of 6.6 mg/kg in pigs 11 days-of-age (21), as well as piglets at 6 and 21 days-of-age receiving an intravenous dose of 6.6 mg/kg (22). However, there have been no pharmacokinetic studies of the EU label dose of 3.0 mg/kg in piglets.

Studies have suggested that plasma drug concentrations do not always reflect tissue drug concentrations, particularly for NSAIDs, which may become “trapped” at sites of inflammation (23–25). A minimally invasive technique (*in vivo* ultrafiltration) collects tissue interstitial fluid (ISF) over time (25, 26). ISF allows the measurement of only the pharmacologically active, protein-unbound drug concentrations, critical to assess drug concentrations directly at the tissue level. It is likely that drug concentrations measured in interstitial fluid will more accurately predict clinical outcome and can be used to make predictions regarding provision of analgesia. The tissue pharmacokinetics of meloxicam were described following an intravenous dose of 0.4 mg/kg in 16–23 day old piglets using a carrageenan-sponge model of acute inflammation (19). However, sampling of tissue fluid using this method collects both protein-bound and unbound drug, which is not representative of the pharmacologically active drug fraction (25). NSAIDs are bound extensively to plasma proteins (generally >95%) (23). As they are weakly acidic drugs, they primarily bind to albumin, which is found in the interstitial space and in sites of inflammation. Protein-bound NSAIDs will become unbound, at which point they can exert their anti-inflammatory activity. The aim of the ultrafiltration probes is to quantify only the protein-unbound portion of each NSAID, as it is anticipated that it will be different from protein-bound, as well as plasma, concentrations. A transudate fluid is generally lower in protein and inflammation, when compared with an exudate, so ultrafiltration probes are likely describing pharmacokinetics in uninflamed transudate, which is likely different to inflamed exudate (27, 28). To date, there are no data available describing the protein-unbound tissue pharmacokinetics of meloxicam, flunixin, or ketoprofen in neonatal piglets, or plasma pharmacokinetic data of these drugs at EU labeled doses in the target age piglet.

This study aimed to assess the plasma and tissue pharmacokinetics of three NSAIDs; meloxicam, flunixin and ketoprofen, and utilized a novel LC-MS/MS method for the enantioselective quantification of ketoprofen in a small sample volume with no derivatization required.

## Materials and Methods

### Animals

Twenty-four Yorkshire/Landrace cross, uncastrated, male piglets from 12 different litters, of 6 ± 1 days of age and weighing 1.92–3.22 kg at the time of dosing, were enrolled as part of a larger study. The piglets were sourced from the North Carolina State University Swine Education Unit and transferred to the North Carolina State University College of Veterinary Medicine where they were housed individually, but able to see one another. Lighting consisted of 12/12 h light/dark, and heat lamps were positioned above the piglets on one end of the individual pens. Ambient room temperature was maintained at 26–30 degrees Celsius. Once removed from the sow, piglets were fed non-medicated swine milk replacer (Milk Specialties Global, Eden Prairie, MN, USA) and offered fresh water every 4 h from 7 a.m. to 12 a.m.

### Catheter and Interstitial Probe Placement

At 4 days-of-age (±1 day), piglets were removed from the sow and moved to individual housing to prevent damage to sampling apparatus. Piglets were anesthetized using sevoflurane (SevoFlo®, Zoetis, Parsippany, NJ) administered in 100% oxygen via face mask. An indwelling jugular catheter (22 Ga, 10 cm small animal long term venous catheterization kit, MILA International, Inc., Florence, KY, USA) was used for collection of blood samples. The catheter was placed percutaneously in the jugular vein using a Seldinger technique similar to previously described Flournoy and Mani (29). The catheter was sutured to the skin near the entry point and covered with a small piece of foam to protect the catheter. An extension set was attached to the catheter and then the neck was wrapped with Ioban to secure it. A small, handmade “pouch” was created using bandage tape and attached to the Ioban at the back of the piglet's neck to store the end of the catheter and allow easy access for sample collection. At the time of IV catheter placement, an ultrafiltration probe (RUF-3-12 Reinforced *In Vivo* Ultrafiltration Sampling Probes, BASi systems, W. LaFayette, IN, USA) was placed subcutaneously along the epaxial muscles using a previously described technique (26, 30). The interstitial probe allowed for continuous collection of interstitial fluid (ISF). Piglets were able to recover for 36–48 h following the placement of instrumentation. During this recovery period, patency of the catheter was maintained by removing the heparin lock, flushing the catheter with saline and replacing the heparin lock every 12 h.

### Drug Administration

At 6 days of age (±1 day) piglets were injected intramuscularly with one of 3 treatments; 0.4 mg/kg meloxicam (Meloxicam solution for injection 5 mg/mL, Putney, Inc., Portland, ME, USA), 2.2 mg/kg flunixin meglumine (Banamine-S®, Merck Animal Health, Summit, NJ, USA) or 3 mg/kg ketoprofen (Ketofen®, Zoetis, Inc., Kalamazoo, MI, USA). Treatment groups were assigned using a random number generator (Microsoft Excel 2016, Microsoft Corporation). The doses were chosen based on existing EU labels for piglets at castration (meloxicam and ketoprofen) or existing USA label dose for other indications in pigs (flunixin). Two hours after drug administration, the piglets were processed (defined in this study as only castrated and tail-docked). Piglets were restrained to expose the anogenital region of the piglet, while a second person performed the procedure. An incision was made on each side of the scrotum using a scalpel, the testicles were pulled from the surrounding tissue and the scalpel was used to cut the testicles free. The tail was then docked using standard tail clippers. Both the castration site and tail were sprayed with betadine to disinfect the wounds.

### Sample Collection

Blood samples (1 mL) were collected and transferred into lithium heparin tubes via the jugular catheter at 0 (baseline), 0.25, 0.5, 1, 1.5, 2, 4, 6, 8, 12, 24, 36, and 48 h after drug administration. Blood samples were centrifuged at 3,500 × g and the plasma collected for analysis of total drug concentrations. Interstitial fluid samples were collected via the preplaced collection probes at 0 (baseline), 2, 4, 6, 8, 12, 24, 36, and 48 h post-dose and weighed to determine the volume collected. At the end of the experiment, the ISF probe was removed and the tubing length measured. A lag time for the ISF collection was calculated to account for the time taken for the sample to travel along the ISF probe tubing (tube length/[total volume collected/total time]). Interstitial fluid was used to quantify the free (protein unbound/pharmacologically active portion) drug concentrations in the tissues. Both plasma and ISF were frozen at −80°C until analysis.

### Analytical Methods

Different analytical methods were used for plasma and ISF due to the small volume of ISF samples. Methods for ISF were developed after the plasma analysis was already completed. For all analytical methods, validation standards were prepared over a linear range for each drug in each matrix (meloxicam, flunixin, and ketoprofen in plasma and ISF) and were used to construct calibration curves. All calibration curves were linear with a *R*^2^ value of 0.99 or higher. Limit of quantification, inter-day accuracy, and inter-day precision are presented in Table 1 for each analytical method.

**Table 1:** Analytical standard concentration range, limit of quantification (LOQ), inter-day accuracy (%), and inter-day precision (%) for analytical methods.

**SAMPLE ANALYSIS PARAMETERS**									
**Drug**	**Matrix**	**Concentration Range**			**LOQ**	**Accuracy (%)**		**Precision (%)**	
		**μg/mL**			**μg/mL**	**Mean**	**±SD**	**Mean**	**±SD**
Meloxicam	Plasma	0.01	–	10	0.01	99	±6	8	±3
	ISF	0.001	–	0.05	0.001	99	±10	2	±2
Flunixin	Plasma	0.0005	–	5	0.0005	103	±7	8	±5
	ISF	0.0005	–	0.2	0.0005	100	±8	3	±2
S(–)-Ketoprofen	Plasma	0.05	–	10	0.05	101	±4	7	±5
	ISF	0.001	–	0.5	0.001	101	±11	7	±6
R(+)-Ketoprofen	Plasma	0.05	–	10	0.05	100	±3	7	±5
	ISF	0.001	–	0.5	0.001	107	±6	6	±3

*SD, standard deviation*.

#### Meloxicam Plasma Analysis

Plasma concentrations of meloxicam were determined using high-performance liquid chromatography (HPLC; 1260 Infinity HPLC system with a multiwavelength detector, Agilent Technologies, Wilmington, DE, USA). The UV detector was set at a wavelength of 365 nm. The column was a 4.6 × 150 mm C18 column (Zorbax SB-C18; MAC-MODAnalytical, Inc., Chadds Ford, PA, USA) kept at a constant temperature of 40°C, and a flow rate of 1 mL/min. Mobile phase consisted of 60% 0.05M sodium acetate buffer (pH 3.75) and 40% acetonitrile (ACN). Meloxicam plasma samples, calibration samples, and blank (control) were prepared using solid phase extraction (1cc Waters Oasis Extraction Cartridges, Waters Corporation, Milford, MA, USA), conditioned with 1 mL methanol followed by 1 mL distilled water. A plasma sample (200 μL) was added to a conditioned cartridge, washed with 1 mL water: methanol (95:5 v/v), and then eluted with 1 mL 100% methanol. Samples were then evaporated at 40°C for 15–20 min. Each sample was then reconstituted with 200 μL of mobile phase and vortexed. Twenty-five microliters were then injected into the HPLC system.

#### Ketoprofen Plasma Analysis

Plasma concentrations of ketoprofen were analyzed using the same HPLC system as the meloxicam plasma samples. For ketoprofen, the UV detector was set at a wavelength of 255 nm. The column was a 4.6 × 150 mm, 5 μm chiral column (Agilent Ultron ES-OVM; Agilent Technologies), kept at 25°C. Mobile phase consisted of 89% 0.02 M potassium monobasic phosphate buffer and 11% ACN. Ketoprofen plasma samples, calibration samples, and blank (control) were prepared using solid phase extraction (3cc Waters Oasis Extraction Cartridges, Waters), conditioned with 1 mL methanol followed by 1 mL distilled water. A plasma sample (200 μL) was added to a conditioned cartridge, washed with 1 mL water: ammonium hydroxide (95:5 v/v), and then eluted with 1 mL methanol:formic acid (98:2). Samples were then evaporated at 30°C for 20–30 min. Each sample was then reconstituted with 200 μL of water and vortexed. Thirty microliters were then injected into the HPLC system. Standards spiked with S(–)-ketoprofen only, were also analyzed at the same time to determine the retention time, allowing separate identification of the S(–)- and R(+)-enantiomer.

#### Flunixin Plasma Analysis

Flunixin plasma concentrations were quantified by ultra-high-pressure liquid chromatography (UPLC) with mass spectrometric (MS/MS) detection (Waters Corp., Milford, MA, USA). The UPLC-MS/MS system consisted of a Xevo TQD tandem quadrupole mass spectrometer (Waters Corp.) For all flunixin sample matrices (plasma and ISF), the UPLC-MS/MS analysis consisted of a 2.1 × 100 mm, 1.8 um HSS T3 column (Waters Corp.) A gradient was used, and the initial mobile phase was 0.1% formic acid in water: 0.1% formic acid in acetonitrile (70:30 v/v) with a flow rate of 0.4 mL/min for the first 2.5 min. The mobile phase then switched to (10:90 v/v) from 2.5–3.5 min. For the last 1.5 min of the run, the mobile phase was (70:30 v/v). The MS/MS was run in ESI+ mode. The quantification trace used was 297 → 279. Column temperature was 35°C and sample temperature was ambient. Flunixin plasma samples were combined with 250 μL 0.5% citric acid in ACN, vortexed thoroughly and then centrifuged for 10 min at 3,000 × g. The supernatant was collected and evaporated at 55°C for 60 min under an 18-psi stream of air. Each sample was then reconstituted with 100 μL of water:ACN (50:50 v/v) and vortexed, filtered through a 0.2 μm filter and then injected.

#### Meloxicam ISF Analysis

Meloxicam ISF concentrations were quantified by UPLC-MS/MS (system information as mentioned previously). UPLC-MS/MS analysis consisted of a 2.1 × 50 mm, 1.7 μm Waters Acquity BEH C18 column (Waters Corp.) A gradient was used, and the initial mobile phase was 0.1% formic acid in water: 0.1% formic acid in acetonitrile (65:35 v/v) with a flow rate of 0.4 mL/min for the first minute. The mobile phase then switched to (10:90 v/v) from 1.0 to 1.1 min. For the last 1.9 min of the run, the mobile phase was (65:35 v/v). The MS/MS was run in ESI+ mode. The quantification trace used was 352.043 → 115. Column temperature was 35°C and sample temperature was 10°C. Fifteen microliters of ISF were combined with 50 μL MeOH, filtered through a 0.2 μm syringe filter and then injected directly onto the chromatography system.

#### Flunixin ISF Analysis

Flunixin ISF samples were analyzed using the same UPLC-MS/MS system as previously described and using the same analytical method as for flunixin in plasma. The flunixin ISF sample preparation was the same as described for meloxicam ISF.

#### Ketoprofen ISF Analysis

Ketoprofen ISF samples were analyzed using the same UPLC system as previously described. The samples were prepared using solid phase extraction. OASIS HLB μElution Plates (Waters, Milford, MA, USA) were used. These were conditioned sequentially with 500 μL of methanol and 500 μL of ultrapure water. Fifty microliters (50 μL) of ISF were loaded into the plate. The loaded plates were washed with 50 μL of 90:10 water: methanol (v/v). Then, the retained ketoprofen was eluted with a total of 50 μL of 80:20 water: acetonitrile (v/v, eluted twice with 25 μL). Five microliters (5 μL) of the eluted solution was directly injected in the UPLC-MS/MS.

The UPLC-MS/MS analysis consisted of a 100 × 3.0 mm, 1.6 μm Chiralpak® IG-U column (Chiral Technologies, Inc., West Chester, PA, USA). A gradient was used, and the initial mobile phase was 0.1% formic acid in water: 0.1% formic acid in methanol (21:79 v/v) with a flow rate of 0.35 mL/min for the first 5.5 min. The mobile phase then switched to (5:95 v/v) from 5.5 to 7.0 min. For the last 2.0 min of the run, the mobile phase was (21:79 v/v). The MS/MS was run in ESI+ mode. The quantification trace used was 255.19 → 104.943, and the R(+)- and S(–)-ketoprofen enantiomers were separated by retention time. Column temperature was 25°C and sample temperature was 15°C.

### Pharmacokinetic Analysis

Pharmacokinetic analysis of drug concentration vs. time profiles was performed with Phoenix WinNonLin software (version 8.0; Certara, Princeton, NJ, USA). A non-compartmental analysis was used to derive the mean residence time (MRT; h), slope of the terminal phase (λz; 1/h), and the half-life (T_1/2_; h). The area under the plasma concentration–time curve from time zero to infinity (AUC0→ ∞; h × μg/mL) was calculated by the linear trapezoidal rule. The volume of distribution (per fraction absorbed) (Vd/F; L/kg) and clearance per fraction absorbed (Cl/F; L/h/kg) were also determined and values for maximum concentration (Cmax; μg/mL) and time to maximum concentration (Tmax; h) were taken directly from the data.

## Results

### Meloxicam

**Figure 1:**
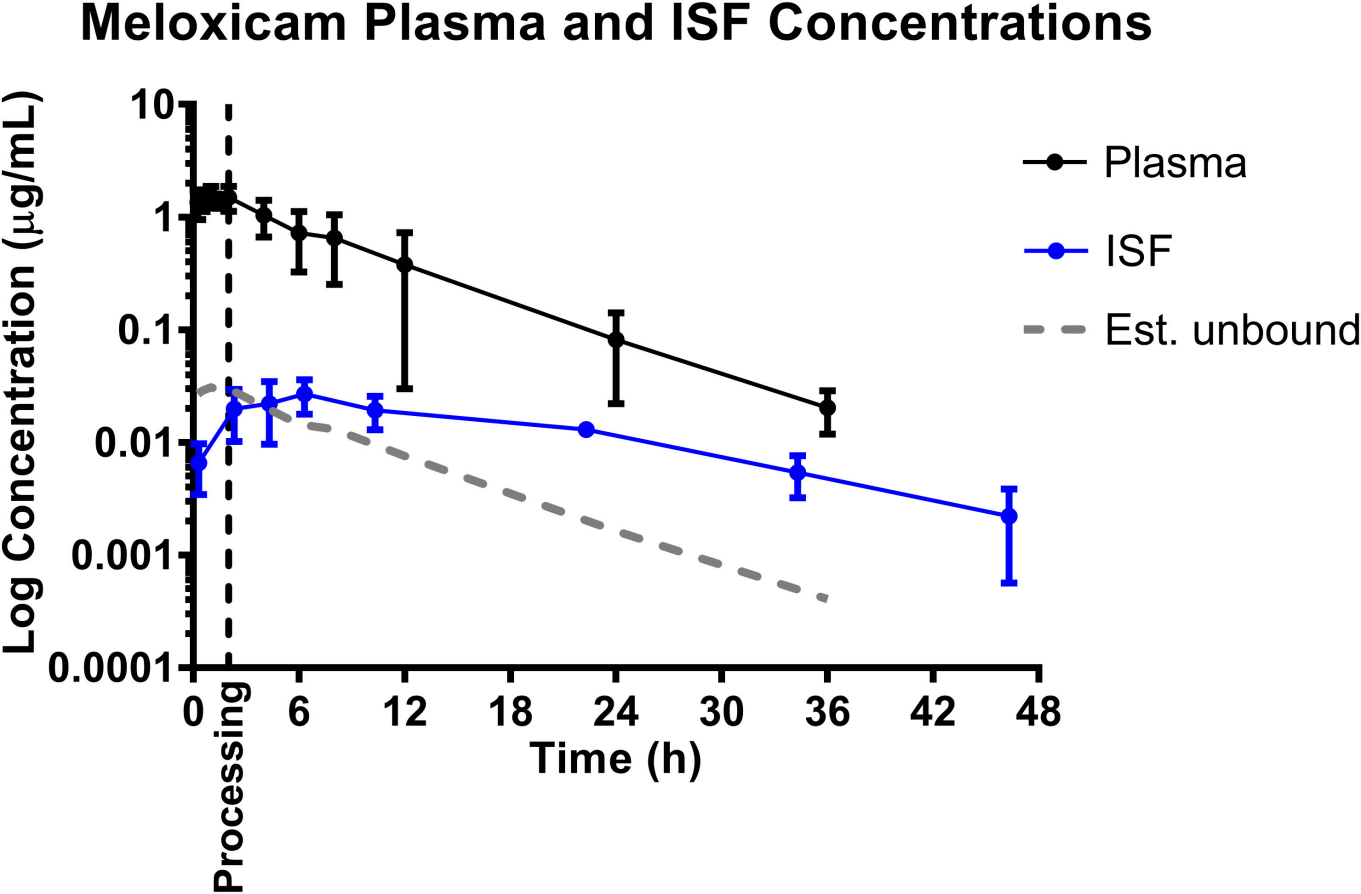
Total plasma concentrations and free/unbound ISF concentration over time following intramuscular injection of 0.4 mg/kg meloxicam in 6-day-old piglets. ISF plotted with lag time (1.68 h). Dose was administered at 0 h and processing (castration and tail-docking) was performed at 2 h, as indicated by the vertical dotted line. Data are represented as mean ± standard deviation. Est. unbound represents the percentage of the total plasma concentration that is estimated to be free or unbound from plasma proteins, assuming meloxicam is normally 98% protein bound (31).

Mean plasma and ISF meloxicam concentrations over time following a single IM injection of 0.4 mg/kg are presented in Figure 1. Parameters describing the plasma pharmacokinetics of meloxicam following a single IM injection are presented in Table 2. Parameters describing the disposition of meloxicam in ISF are presented in Table 3. Meloxicam concentrations in plasma fell below the LOQ of 0.01 μg/mL after 36 h in all piglets. Meloxicam was still detected above the LOQ of 0.001 μg/mL in ISF at the end of the study (48 h). The plasma pharmacokinetics of meloxicam after IM administration were characterized by rapid absorption and a brief apparent terminal half-life (4.46 ± 1.52 h), compared to ISF in which meloxicam persisted for a longer time (apparent half-life 11.26 ± 4.15 h).

**Table 2:** Non-compartmental plasma pharmacokinetic parameters following intramuscular administration of NSAIDs (meloxicam 0.4 mg/kg, flunixin 2.2 mg/kg, and ketoprofen 3 mg/kg) to 6-day-old piglets.

**PLASMA PHARMACOKINETIC PARAMETERS**							
**Parameter**	**Units**	**Meloxicam**		**Flunixin**		**S(–)-Ketoprofen**	
Dose	mg/kg	0.40		2.20		3.00	
MRT	h	5.80	(2.23)	9.77	(3.64)	5.11	(1.19)
T_1/2_	h	4.46	(1.52)	7.93	(2.91)	3.50	(0.80)
λ_z_	1/h	0.18	(0.09)	0.10	(0.04)	0.21	(0.05)
T_max_	h	1.21	(0.68)	0.85	(0.70)	0.59	(0.27)
C_max_	μg/mL	1.58	(0.34)	3.94	(0.86)	9.13	(1.75)
AUC_last_	h.μg/mL	10.34	(3.97)	27.25	(9.06)	52.26	(14.61)
AUC_inf_	h.μg/mL	10.75	(4.41)	28.06	(9.91)	53.74	(14.79)
AUC_extrap_	%	3.75	(4.41)	2.35	(2.91)	2.82	(2.30)
Vd/F	L/kg	0.24	(0.04)	0.92	(0.21)	0.29	(0.04)
Cl/F	L/h/kg	0.04	(0.02)	0.09	(0.03)	0.06	(0.02)

*Data are shown as mean (standard deviation)*.

**Table 3:** Non-compartmental ISF pharmacokinetic parameters following intramuscular administration of NSAIDs (meloxicam 0.4 mg/kg, flunixin 2.2 mg/kg, or ketoprofen 3.0 mg/kg) to 6-day-old piglets.

**ISF PHARMACOKINETIC PARAMETERS**							
**Parameter**	**Units**	**Meloxicam**		**Flunixin**		**S(–)-Ketoprofen**	
Dose	mg/kg	0.40		2.20		3.00	
MRT	h	16.23	(5.00)	24.24	(9.98)	8.57	(2.05)
T_1/2_	h	11.26	(4.15)	16.34	(7.09)	5.54	(0.99)
T_max_	h	2.81	(1.00)	3.64	(1.63)	2.98	(1.03)
C_max_	μg/mL	0.032	(0.004)	0.024	(0.009)	0.300	(0.079)
AUC_last_	h.μg/mL	0.537	(0.240)	0.476	(0.211)	3.058	(0.669)
AUC_inf_	h.μg/mL	0.613	(0.216)	0.577	(0.295)	3.081	(0.672)
AUC_extrap_	%	15.86	(16.41)	14.18	(10.07)	0.73	(0.72)

*Data are shown as mean (standard deviation)*.

### Flunixin

**Figure 2:**
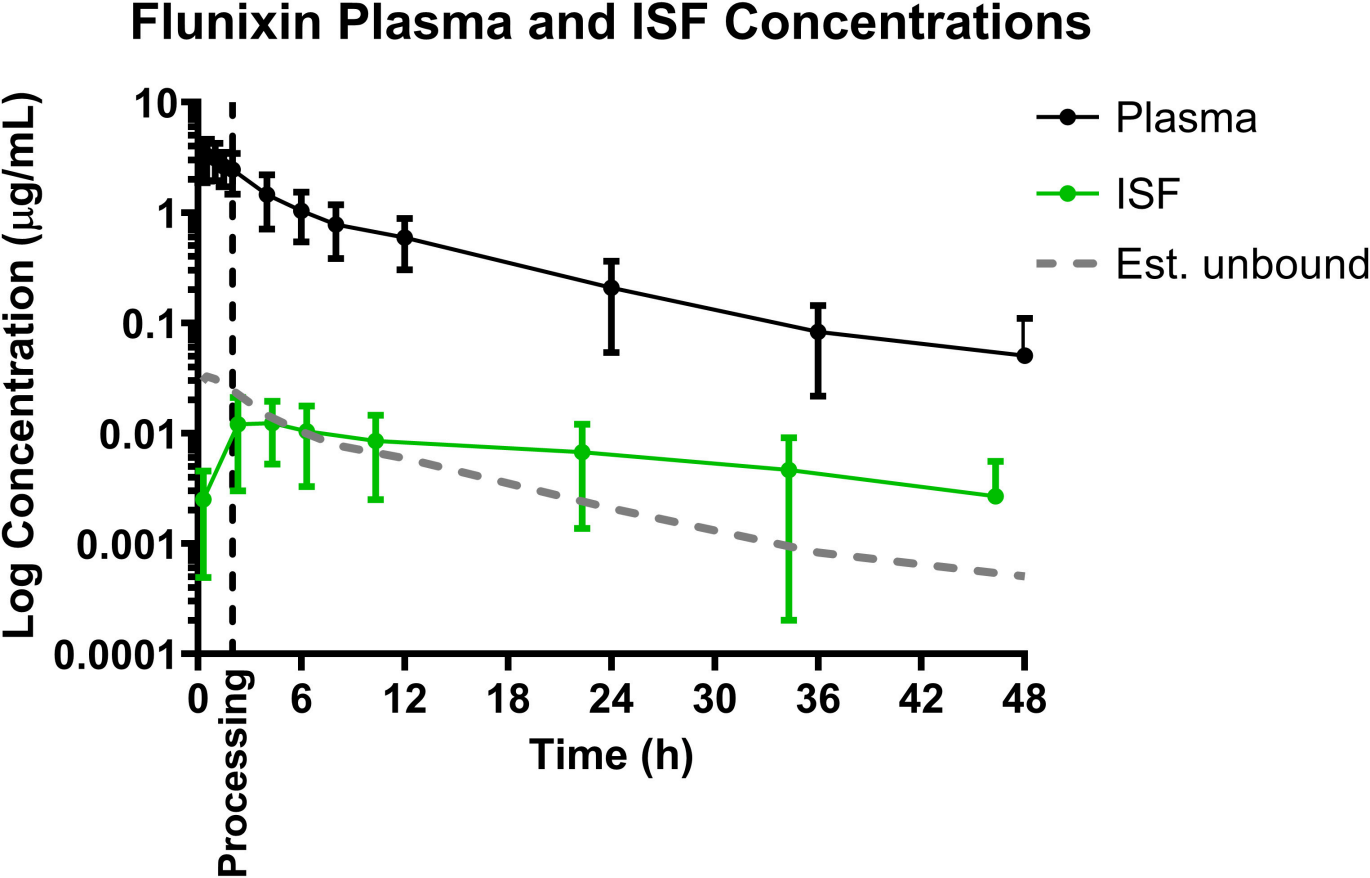
Total plasma concentrations and free/unbound ISF concentration over time following intramuscular injection of 2.2 mg/kg flunixin in 6-day-old piglets. ISF plotted with lag time (1.68 h). Dose was administered at 0 h and processing (castration and tail-docking) was performed at 2 h, as indicated by the vertical dotted line. Data are represented as mean ± standard deviation. Est. unbound represents the percentage of the total plasma concentration that is estimated to be free or unbound from plasma proteins, sassuming flunixin is normally 99% protein bound (32).

Mean plasma and ISF flunixin concentrations over time following a single IM injection of 2.2 mg/kg are presented in Figure 2. Parameters describing the plasma pharmacokinetics of flunixin following a single IM injection are presented in Table 2. Parameters describing the disposition of flunixin in ISF are presented in Table 3. Flunixin concentrations in both plasma and ISF were still detected above the LOQ of 0.0005 μg/mL at the end of the study (48 h) in all piglets. The plasma pharmacokinetics of flunixin after IM administration were characterized by rapid absorption, large volume of distribution/F (0.92 ± 0.21 L/kg) and an apparent terminal half-life (7.93 ±2.91 h) which was short compared to ISF in which flunixin persisted for a longer time (apparent half-life 16.34 ± 7.09 h).

### Ketoprofen

Mean plasma and ISF ketoprofen concentrations over time following a single IM injection of 3.0 mg/kg are presented in Figure 1. Parameters describing the plasma pharmacokinetics of ketoprofen following a single IM injection are presented in Table 2. Parameters describing the disposition of ketoprofen in ISF are presented in Table 3. Ketoprofen concentrations in plasma fell below the LOQ of 0.05 μg/mL after 24 h in all piglets for both enantiomers. S(–)- and R(+)-ketoprofen were still detected above the LOQ of 0.001 μg/mL in ISF at the end of the study (48 h). The plasma pharmacokinetics of S(–)-ketoprofen after IM administration were characterized by rapid absorption and a short apparent terminal half-life (3.50 ± 0.80 h), as well as a relatively short apparent half-life in ISF (5.54 ± 0.99 h). Unfortunately, pharmacokinetic parameters could not be calculated for R(+)-ketoprofen. However, the plasma pharmacokinetics were characterized by rapid decrease in concentration in a short amount of time [R(+)-ketoprofen was last detected at 4 h after administration of the dose], compared to ISF in which the concentration of R(+)-ketoprofen persisted for much longer.

## Discussion

Following intramuscular administration of 0.4 mg/kg meloxicam, the peak plasma concentration was reached in 1.21 h, which was delayed compared to the previously reported 0.50 h in 8-day-old piglets given an intramuscular dose of 1 mg/kg (17). However, the apparent terminal half-life was comparable to the previous report [4.46 h in this study compared to 3.94 h; (17)]. Both of these values in piglets are shorter than 6.15 h, which is the terminal half-life reported in mature sows following an intravenous dose of 0.5 mg/kg (33). While these studies used different doses and routes of administration, this difference in half-life may suggest that drug elimination in piglets may be more rapid than that of mature pigs, which could be clinically important in the duration of analgesia provided post-processing. However, given that terminal half-life is a hybrid parameter that incorporates both volume of distribution and clearance, mechanisms for differences in plasma terminal half-life remain unknown at this time. Typically, the neonate has reduced clearance of many drugs compared with older individuals largely due to the greater body water content leading to a higher volume of distribution, a larger fraction of body mass that consists of highly perfused tissues, a lower plasma concentration of proteins that bind drugs and incomplete maturation of their hepatic-enzymes systems. Differences in sampling and analytical methodologies between studies could account for some of these differences, but without a direct comparison, it is unclear.

Following intramuscular administration of 2.2 mg/kg flunixin, the peak plasma concentration was reached in 0.85 h, which is comparable to the previously reported Tmax of 0.61 h following intramuscular administration to gilts (34). The apparent terminal half-life was also comparable [7.93 h in this study compared to 7.49 h (34)]. However, both of these were longer than previously reported in 10-day-old piglets following intravenous administration of 2.2 and 4.4 mg/kg flunixin [4.82 and 5.15 h, respectively (20)]. This is likely due to the different route of administration, as absorption continues following intramuscular administration while elimination is occurring, compared to intravenous that does not require absorption.

Following intramuscular administration of 3.0 mg/kg racemic ketoprofen, the peak plasma concentration of S(–)-ketoprofen was reached in 0.59 h, which was the most rapid of the NSAIDs investigated in this study, and was comparable to the previously reported Tmax of 0.68 h in 11 day old piglets given an intramuscular dose of 6.6 mg/kg (21). The apparent terminal half-life was almost identical to that previously reported [3.50 h in this study compared to 3.51 h (21)], and was similar to reports following intravenous doses of 6.6 mg/kg in piglets 6 and 21 days-of-age [3.4 and 3.3 h, respectively (22)]. The plasma concentration-time profile was very similar to that of 11-day-old piglets given 6.6 mg/kg IM, including R(+)-ketoprofen which rapidly decreased in concentration and was last detected by 4 h after administration of the dose in both studies.

Volume of distribution per fraction absorbed (Vd/F) in this study for meloxicam was comparable to that of other studies investigating piglets 8–23 days of age, given doses in the range of 0.4–1.0 mg/kg and given via intramuscular or intravenous routes of administration (17, 19, 20). S(–)-ketoprofen also had a comparable Vd/F (0.29 L/kg) compared to previous reports following intramuscular administration of 6.6 mg/kg in piglets 8–17 days-of-age [0.30 L/kg (21)]. There are no reports of volume of distribution/F following an intramuscular dose for flunixin, but the value reported in the present study (0.92 L/kg) is greater than that previously reported following IV doses in 10-day-old piglets [0.25 L/kg and 0.26 L/kg, (20)]. However, it is also much lower than that reported in juvenile pigs (18–27 kg body weight) following IV dose [1.83 L/kg, (32)]. In gilts, bioavailability of flunixin has been reported at 76% following an intramuscular dose of 2.2 mg/kg compared to an IV dose of the same amount (34), but there have been no bioavailability studies in piglets. These differences could be due to differences in the route of administration (volume of distribution at steady state compared to volume of distribution not corrected for bioavailability), differences in early sampling time points or modeling methods, and/or differences in total body water composition as a result of age differences. Interestingly, flunixin had the longest apparent terminal half-life of the three NSAIDs studied, despite also displaying the highest clearance/F. This demonstrates the effect of volume of distribution on half-life, as flunixin also demonstrated the largest Vd/F in this study, which may indicate greater tissue penetration and presence at the site of action, although this cannot be confirmed without bioavailability data for these NSAIDs in this population of piglets.

Overall, plasma NSAID concentrations did not predict or reflect the tissue concentration data. For example, the time to maximum concentration and half-life were longer in the tissues as demonstrated by the ISF data. Although maximum concentrations were lower in the tissues, these data tentatively reflect only plasma unbound drug concentrations. These plasma unbound concentrations are more pharmacodynamically relevant, and are expected to be lower, because most NSAIDs are highly protein bound (generally > 95%) (23).

**Figure 3:**
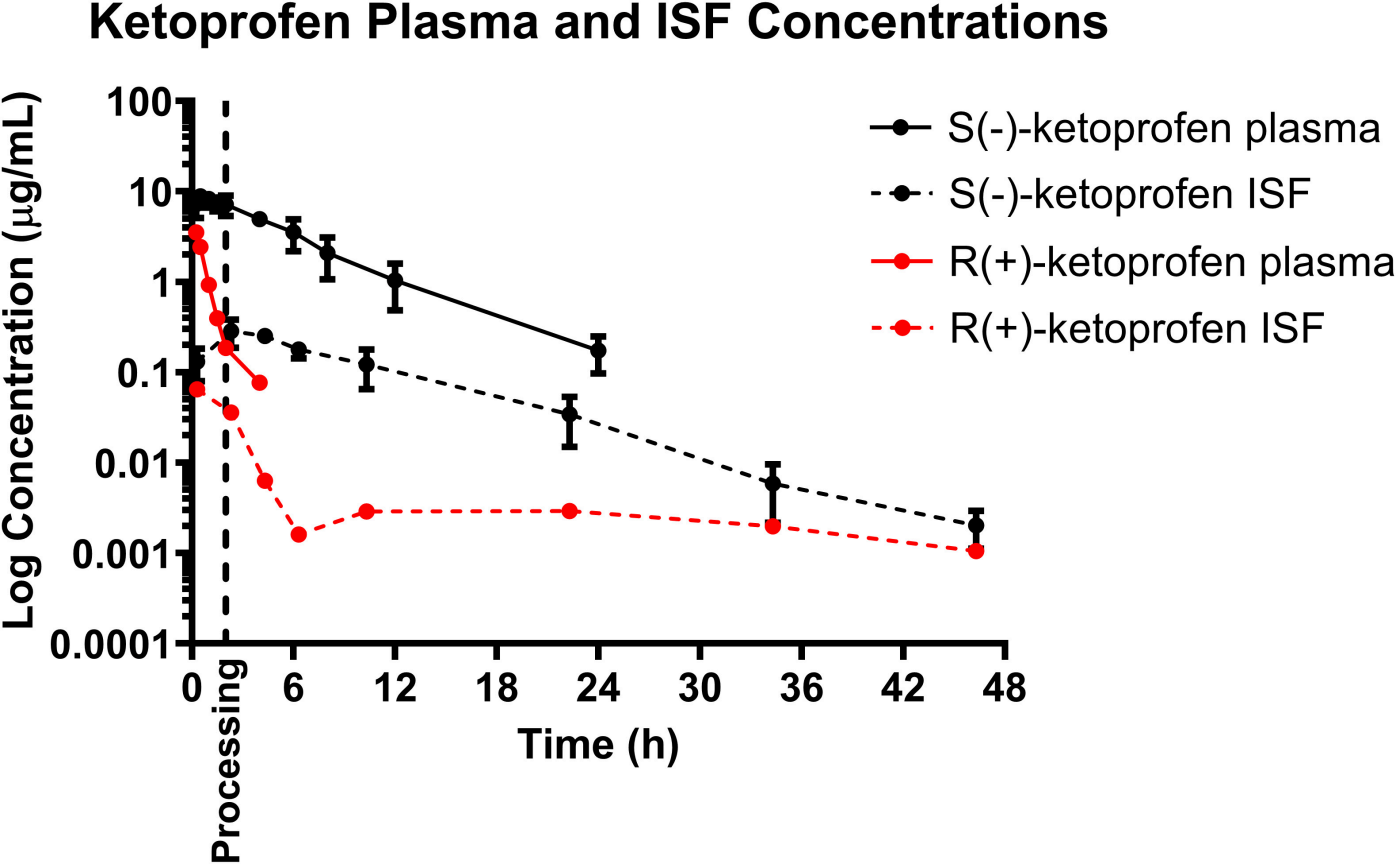
Total plasma concentration over time following intramuscular injection of 3 mg/kg ketoprofen in 6-day-old piglets. Both the S(–)- and R(+)-enantiomers of ketoprofen are shown. Dose was administered at 0 h and processing (castration and tail-docking) was performed at 2 h, as indicated on the plot by a vertical dotted line. Data are represented as mean ± standard deviation. Protein binding has not been measured for the separate enantiomers so estimated unbound concentration in the plasma has not been shown in this figure.

Based on the tissue (ISF) pharmacokinetic data, administration of each NSAID 2 h prior to castration and tail-docking was an ideal time to administer these drugs as maximum tissue concentrations were achieved within 2–4 h of administration. NSAIDs are highly protein bound in the plasma, so tissue concentrations of drugs are more likely to be representative of the effective concentration at the site of action. As can be seen in Figures 1–3, tissue concentrations of both meloxicam, flunixin and S(–)-ketoprofen were detected at the last time point assessed (48 h). These results are different from those previously reported for meloxicam tissue exudate, which reported concentrations only to 12 h (19). Higher concentrations were reported, which is likely reflective of the sampling methodology which was a tissue cage vs. an ultrafiltration probe. However, care should be taken when comparing pharmacokinetic parameters or concentrations between plasma and ISF in this study, due to the different limits of quantification for each assay. This is because new methods for ISF were developed due to the small sample volume, and these methods were developed after the plasma analysis was already completed.

**Figure 4:**
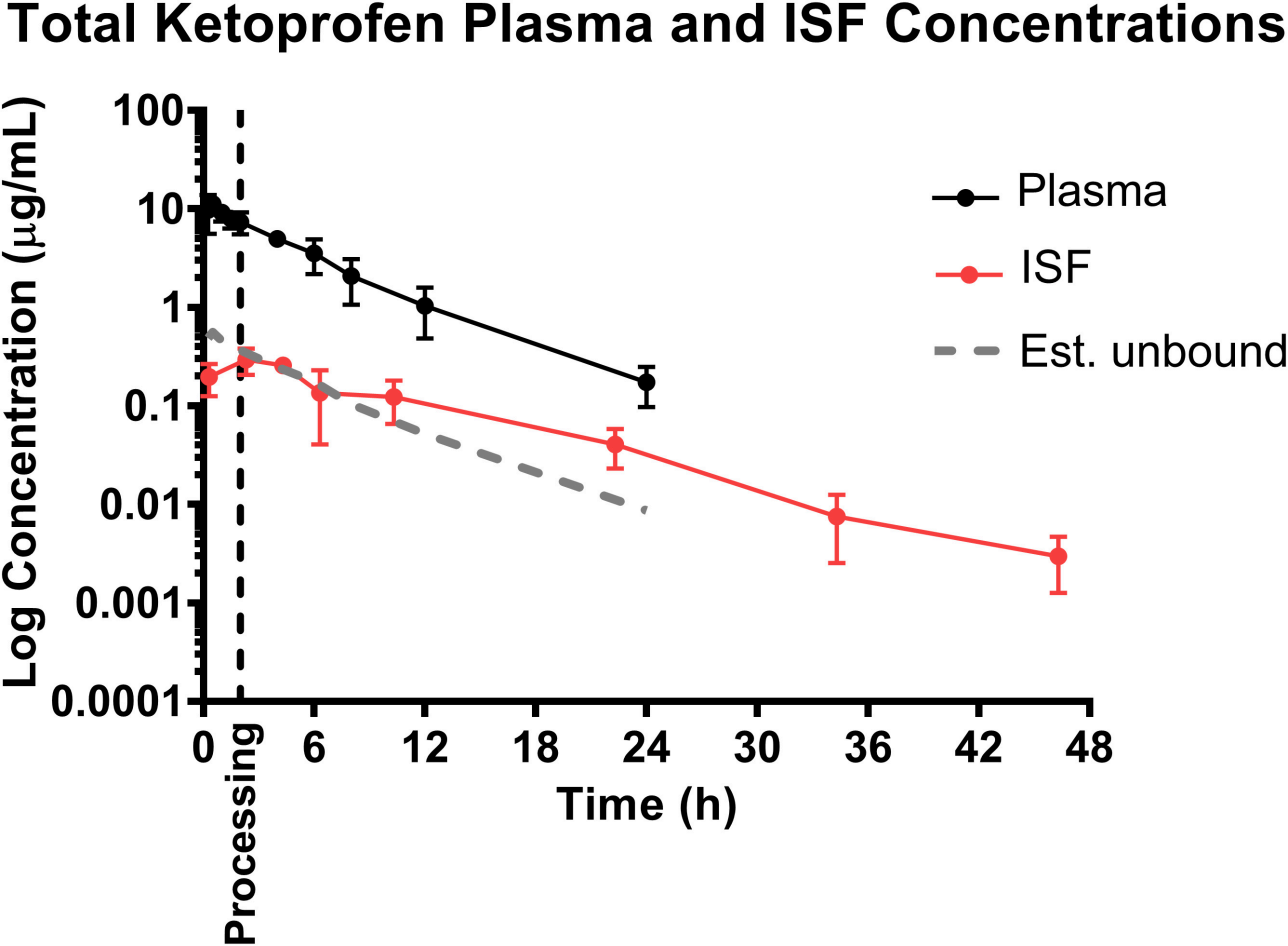
Total ketoprofen plasma concentration over time following intramuscular injection of 3 mg/kg ketoprofen in 6-day-old piglets [sum of both S(–)- and R(+)-ketoprofen]. Dose was administered at 0 h and processing (castration and tail-docking) was performed at 2 h, as indicated on the plot by a vertical dotted line. Data are represented as mean ± standard deviation, and total ketoprofen was calculated by addition of the two ketoprofen enantiomers. Est. unbound represents the percentage of the total plasma concentration that is estimated to be free or unbound from plasma proteins, assuming ketoprofen is normally 95% protein bound (36).

The ability to study the distribution and anti-inflammatory effects of NSAIDs directly at sites of action (in this case, at the tissue level) can improve understanding of drug effects and allow the application of appropriate dosage regimens (23, 25, 35). Specifically, this knowledge is important when assessing NSAIDs, as plasma drug concentrations have not been correlated with therapeutic efficacy (24). Interstitial fluid can be analyzed for only the pharmacologically active, protein-unbound drug concentrations directly at the tissue level. In addition, unbound drug concentrations can be correlated with objective biomarkers of inflammation in the future, such as prostaglandin E2, thereby establishing therapeutic drug concentrations directly at the effect site. As seen in Figures 1, 2, 4, the concentrations of *estimated* unbound plasma concentrations (dotted line) do not reflect those seen at the tissue level (ISF). Specifically, tissue depletion of NSAIDs tends to be slower. Therefore, measuring unbound concentrations found in plasma may not be appropriate to estimate the pharmacodynamic effects of NSAIDs, and unbound tissue concentrations may correlate better with therapeutic efficacy.

It is additionally important to consider the stereoselective pharmacokinetics of ketoprofen, rather than simply total ketoprofen concentration, as plasma concentration and pharmacodynamic effect of each enantiomer differs between species (37). Previous studies in pigs have reported that the S(–)-enantiomer is a more potent cyclooxygenase-inhibitor, and therefore displays greater anti-inflammatory effects compared to the R(+)-enantiomer (22, 38). However, the R(+)-ketoprofen enantiomer may be a more potent analgesic according to a study assessing mechanical nociceptive threshold (21). In many species, S(–)-ketoprofen predominates over R(+)-ketoprofen in terms of plasma exposure following intramuscular administration of racemic ketoprofen (22, 37–39). The current study also found this to be true in piglets, and additionally found that this was also true for interstitial fluid exposure. Further investigation of the activity of the S(–)- and R(+)-enantiomers will attempt to elucidate the anti-inflammatory and analgesic effect in relation to both the plasma and tissue pharmacokinetics.

When making comparisons between pharmacokinetic parameters of each NSAID, care should be taken as these were given at different doses. However, this information is still important as the doses given were clinically relevant doses that are already being used in practice in the EU, USA or Canada. Secondly, a limitation of the study lies in the comparisons of volume of distribution and clearance are on the basis of “per fraction absorbed,” as the drugs were administered intramuscularly and bioavailability in this age piglet is unknown.

This study was the first to describe both the plasma and tissue pharmacokinetics of each NSAID in the intended population of animals: 6-day old piglets undergoing surgical castration and tail docking. The plasma pharmacokinetic results are comparable to previous reports on pharmacokinetics of meloxicam, flunixin and ketoprofen in piglets of similar age or older, although across studies the routes of administration, doses, and methods of pharmacokinetic analysis differ slightly. This study is the first to report on the tissue pharmacokinetics of each of these drugs in piglets, using a novel, minimally invasive sampling technique of *in vivo* ultrafiltration, and demonstrated not only the feasibility of this technique in neonatal piglets for the first time, but the differences in tissue pharmacokinetics compared to plasma pharmacokinetics for each NSAID. Future studies are currently aimed at establishing a relationship between the ISF concentration-time profiles with pain alleviation.

## Data Availability Statement

The datasets generated for this study are available on request to the corresponding author.

## Ethics Statement

The animal study was reviewed and approved by North Carolina State University Institutional Animal Care and Use Committee.

## Author Contributions

EN contributed to sample collection, sample analysis, pharmacokinetic and statistical analysis, and writing the manuscript. GA and RB contributed to study design and editing the manuscript. KM contributed to study design, sample collection, and editing the manuscript.

### Conflict of Interest

KM has received honoraria and grants or research contracts from the following: Zoetis, Aratana, Bayer, Jurox, Piedmont Animal Health, Mallinckrodt, Innovate Biopharmaceuticals, and RxActuator. The remaining authors declare that the research was conducted in the absence of any commercial or financial relationships that could be construed as a potential conflict of interest.
